# What Is Cholera?

**Published:** 1893-07

**Authors:** William Watson Hall


### WHAT IS CHOLERA?

A very great deal has been uselessly written for public perusal about the causes of cholera. One person will tell you that a glass of soda gave him cholera, or a mess of huckleberries, or cucumbers, or green corn, or cabbages, which is just about as true as the almost universal error, that a bad cold causes consumption. A bad cold never did nor ever can originate consumption, any more than the things above named originate cholera.

A bad cold excites consumption in a person whose lungs are already tuberculated, not otherwise, certainly; and so green corn or cucumbers, or cabbages, or *any other food whatever* it may be, which is not well digested when it passes into the stomach, will excite cholera when a person is living in a cholera atmosphere, and the atmosphere is made "choleraic" by its holding in suspension some emanation which is the product of vegetable decomposition.

LIMESTONE WATER.

Much has been written about this agent as a cause of cholera. Those who know least are most positive. It may be true to some extent, and, under some circumstances it may be an excitant of cholera, but I cannot think it is "per se," that it is remarkably or necessarily so. It is known that the whole south and west has suffered cholera in the past. New Orleans especially; yet there is scarcely a decent dwelling there which has not a cistern attached to it, *above ground*, and wholly supplied by rain water; and this is the usual drink, and it is the same case with multitudes of the better class of dwellings in the southern country. As to escaping prevalent cholera, the great general rules are:

First. Make no violent changes in your mode of life, whether in eating or drinking, or sleeping or exercise.

Second. Endeavor to attain composure of mind, quietude, regularity of all bodily habits, temperance in the use of plain, substantial, nourishing food; and let your drinks be a moderate amount of tea and coffee and cold water. If accustomed to use wine or brandy, or any other beverage, or alcoholic stimulants, make no change, for change is death.

If any change at all, it should be a regular, steady, systematic increase. But as soon as the cholera has disappeared drink no more.

Fruits in cholera times are beneficial if properly used. They should be ripe, fresh, perfect, should be eaten alone without cream or sugar, and without fluids of any kind for an hour after, and they should not be eaten later in the day than the usual dinner hour of two. In cholera times nothing should be taken after dinner, except a piece of cold bread and butter and a cup of tea of some kind. This, indeed, ought to be the rule for all who wish to live long and healthfully. The indefinite unpleasantness in the bowels, which I have so much insisted upon as the real, premonitory symptoms of Asiatic cholera, began, whether there be looseness or constipation, most probably precedes every acknowledged attack of cholera, from hours up to days. There are no means for proving this, certainly; for the mass of people are too unobserving. But it most certainly is a safe rule in cholera times to regard it as premonitory, and to act accordingly.

Whatever I have said of cholera in the preceding pages, I wish to be understood as applicable to what has come under my own observation during the general prevalence of cholera in a community.

In different states and countries there are circumstances which modify the disease, its symptoms and everything connected with it, such as locality, variety of exciting causes, their different degrees of virulence or concentratedness, the different habits and modes of life.

These things constitute the reason of the various modes of treatment, and the great error has been the publishing of a successful remedy in one locality and relying upon it in another. But the treatment of quietude, ice and calomel, is equally applicable in every spot of the earth's surface wherever a case of epidemic cholera occurs, since the essential cause of cholera is everywhere the same, to wit: a failure on the part of the liver to work with sufficient vigor to withdraw the bile from the blood and pass it out of the system; and the mode of removing that effect is the same, to wit, the stimulation of the liver to increased action. And although, in milder forms, a variety of agencies may stimulate the liver to work, and thus restore health, yet inasmuch as calomel is infinitely more reliable than all other liver stimulants yet known, it is recommended as having precedence of all others on the ground previously named, that when danger is imminent and a few hours makes the difference between life and death, it is unwise to trust to a less certain agent when the more certain one is equally at hand, and is the easiest medicine known to be taken, as it has no appreciable taste, its bulk is exceedingly small, and by reason of its weight it sinks to the bottom of the stomach and cannot be rejected except in rare instances.

If, then, calomel is such an admirable agent in cholera, why is it not universally used? I might as well ask if honesty is the best policy, why are not the majority of men honest from principle? It is because men are ignorant or misinformed.

Many persons do not know the power of calomel in curing cholera, while others are afraid of it because it sometimes salivates. Suppose it does, better to run the risk of salivation than die.

And even if salivated, a man is not necessarily permanently injured by salivation. I have been badly salivated several times, very many years ago, but I believe I have as good health as most men.

I do not recollect to have lost three meals from sickness in fifteen years past, except from seasickness, and no doubt there are tens of thousands of persons who have been salivated can speak similarly.

But the objection is perfectly childish when it is remembered that perhaps a thousand persons in succession may take calomel and not two in the thousand be salivated. I might say not two in ten thousand and that in a vast majority of those who are not designedly salivated, this salivation is the result of injudicious administration ; thus—

Salivation is caused by keeping the system too long under the influence of calomel, in two ways. First, by giving small doses at short intervals ; second, by giving an amount so small that it fails to work itself off in ten or twelve hours ; third, by giving a larger amount, but mixing opium in some form or other with it, for in all cases the more opium or other anodyne you give with a dose of calomel, the longer it will be in producing its legitimate action. The best method of administering calomel is to give enough at one time to make it act of itself within twelve hours, and if it does not act within that time, take an injection of half a pint of tepid water, or of a tablespoonful of salts in a half-pint of warm water every hour until the bowels act. Any action of the bowels at all after six hours since taking the calomel may be set down as an action from calomel, and nothing need be done to "work it off."

If salivation is not designed, it is not best to give a dose of calomel oftener than once a week. By observing the two rules just stated, I do not believe that any general practitioner will have one case of undesired salivation in ten years practice. It is important for the reader to remember that there are sporadic cases, that is, scattering cases of cholera which may not be preceded by constipation, or looseness in the bowels, or uneasiness sufficiently decided to have attracted the observation of the patient ; for in many cases the patient declares that he "*felt*" as well as he ever did in his life, or acquaintances remark that he "*appeared*" to be in perfect health, and yet to-day he is dead of cholera. Yet, I very much doubt if a case of cholera ever occurred without the premonitions above named in a greater or less degree. Still, for all practical purposes, and to be on the safe side, let no one who has looseness in cholera times conclude that it cannot be cholera because he "felt" so and so the day before, or because no premonitions were observed ; rather let him conclude they were slight or unobserved and act as he should do if he were perfectly assured that he had at that moment in his own person undisputed epidemic Asiatic cholera. The truth is it is as impossible for a man in perfect health to be stricken down in a moment with a dangerous disease, as it is for a man who has been honest from principle for a lifetime to become in a day a forger or a swindler. As far as my observation has extended, I

believe that the most frequent of all exciting causes of cholera is going to bed too soon after a hearty meal, whether it be a late dinner or merely a supper of fruits and cream, or milk with sugar. I think that eating freely of fruits or berries, ripe and perfect, with any fluid after them and then going to bed in an hour or two, will excite cholera in cholera times. I am inclined to think that huckleberries with cream or milk, except in very small quantity, makes a dangerous diet in cholera times.

I will close the subject with answering an inquiry which no doubt has occurred to the reader, as a conclusive refutation of all that I have said as to the fundamental cause of cholera, to wit: If cholera is the result of heat, moisture, and vegetable matter in combination, why has it not prevailed from time immemorial? Because the climates of the world and of the various countries of the earth are constantly changing, hence new diseases are making their appearance from time to time, while others have vanished from the world. And when a single element of many is changed, an entirely new combination may be the result.

But whatever may be that new or changed element, it can no more, as far as our present knowledge extends, excite epidemic cholera without the aid of vegetable decomposition, than powder can be ignited without the aid of fire. WILLIAM WATSON HALL, M. D.

